# Occult Caries: The Hidden Truth

**DOI:** 10.5005/jp-journals-10005-1083i

**Published:** 2010-09-15

**Authors:** V Satish, Prabhadevi CM, Hegde KV

**Affiliations:** 1 Reader, Department of Pedodontics and Preventive Dentistry, Jaipur Dental College, Jaipur, Rajasthan, India; 2 Senior Lecturer, Department of Pedodontics and Preventive Dentistry, Jaipur Dental College, Jaipur, Rajasthan, India; 3 Reader, Department of Pedodontics and Preventive Dentistry, Jaipur Dental College, Jaipur, Rajasthan, India

**Keywords:** Occult caries, Pre-eruptive carious lesions.

## Abstract

The term “occult caries” and “hidden caries” are used to describe such lesions, which are not clinically diagnosed but detected only on radiographs.

A female patient aged 14 years reported for routine dental check-up. On Intraoral examination, sinus was seen in the buccal mucosa of maxillary left second premolar 25. On clinical examination the occlusal surface remained ostensibly intact; tooth was asymptomatic with no past history of pain. On radiographic examination, there was radiolucency in distobuccal aspect of 25 involving enamel, dentin and nearing pulp with presence of open apex and periapical radiolucency irt 25. With clinical and radiographic evaluation, the case was diagnosed as occult caries in relation to 25. Apexification was done with calcium hydroxide. The tooth is under observation; once radiographic barrier is formed conventional root canal treatment will be performed.

**Conclusion :** Early diagnosis of occult lesions is the best management. As radiographs are probably the most effective method of diagnosing all occult lesions, they should be recommended at appropriate ages to aid early detection of these lesions.

## INTRODUCTION

Dental caries remains a critical concern today and continues to be the most prevalent infectious disease in the world. The last century has provided tremendous advancements in the understanding and treatment of dental caries, yet the disease prevails as a worldwide epidemic. Just our plain understanding that the infectious bacteria will produce acid when supplied with nutrients and cause demineralization of the host tooth surface will not be sufficient to eliminate the caries disease process.

For several decades, pediatric dentists have recognized that there exist some deep occlusal lesions in premolar and molar teeth, which have missed clinical detection because the occlusal surfaces remained ostensibly intact until large parts of the crowns have been destroyed. The term “occult caries” and “hidden caries” are used to describe such lesions, which are not clinically diagnosed but detected only on radiographs.^1^ However, the nature of these lesions, their etiologies, and reasons as to why they have eluded early, clinical diagnosis are still unclear.

## CASE REPORT

**Fig. 1:**
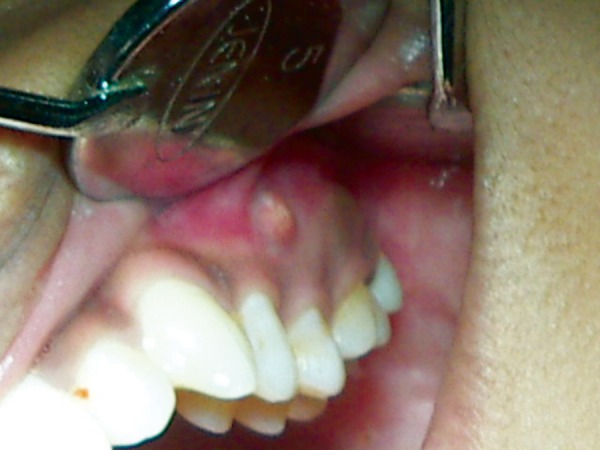
Intraoral sinus in relation to 25

**Fig. 2:**
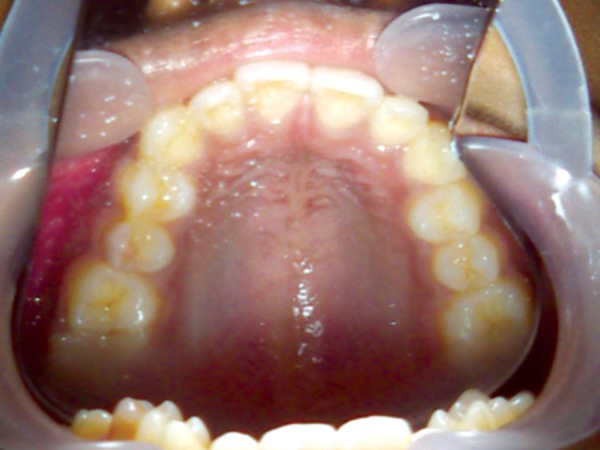
Occlusal view of the maxilla with intact occlusal surface of 25

**Fig. 3:**
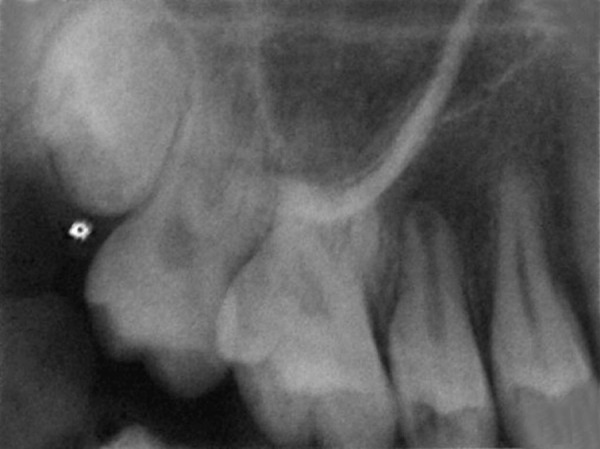
IOPA of 25 revealing the disto-occlusal caries involving enamel and dentin

**Fig. 4:**
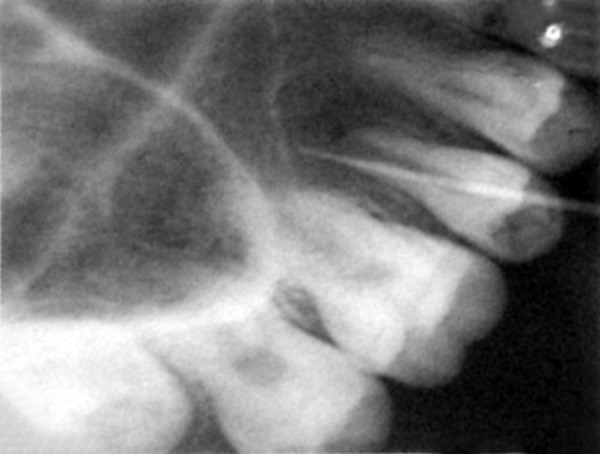
GP point locating sinus pathway

A female patient aged 14 years reported for routine dental check-up. Her medical history and past dental history were noncontributory with no adverse habits. Patient was well built and well nourished with normal vital signs. Face was bilaterally symmetrical with straight profile and competent lips. Lymph nodes were nontender. On intraoral examination, the floor of the mouth, palate was normal and generalized inflammation of marginal gingival was seen. Striking feature was the presence of sinus in the buccal mucosa of maxillary left second premolar 25 (Fig. 1). On examination, the occlusal surface remained ostensibly intact; (Fig. 2) tooth was asymptomatic with no past history of pain. On radiographic examination there was radiolucency in distobuccal aspect of 25 involving enamel and dentin nearing pulp with presence of open apex and periapical radiolucency irt 25 (Fig. 3). To locate the path of the sinus, gutta-percha point was placed in the sinus tract and an intra oral periapical radiograph was taken. The sinus tract was coinciding with the root of 25 indicating the involvement of abscess in relation to 25 region (Fig. 4). With clinical and radiographic evaluation, the case was diagnosed as occult caries irt to 25.

**Fig. 5:**
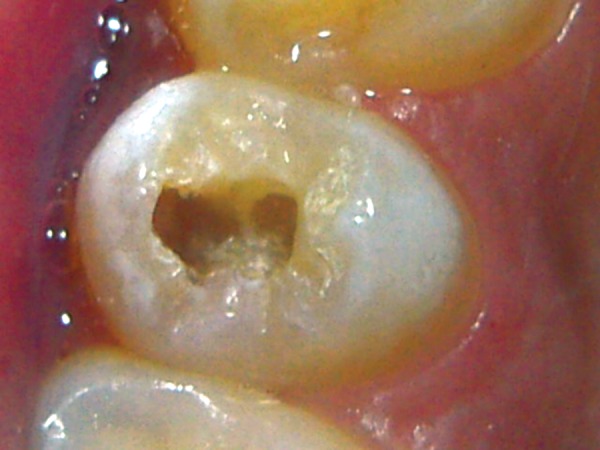
Excavation of occult caries in relation to 25

**Fig. 6:**
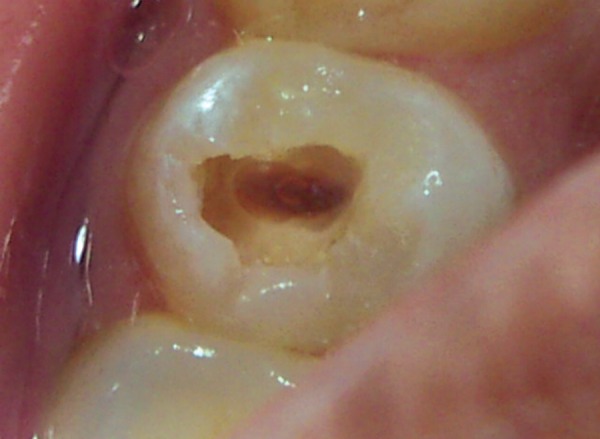
Access opening in relation to 25

**Fig. 7:**
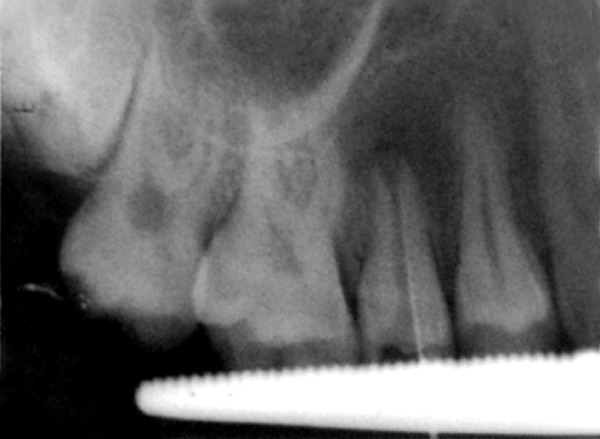
Working length IOPA in relation to 25

**Fig. 8:**
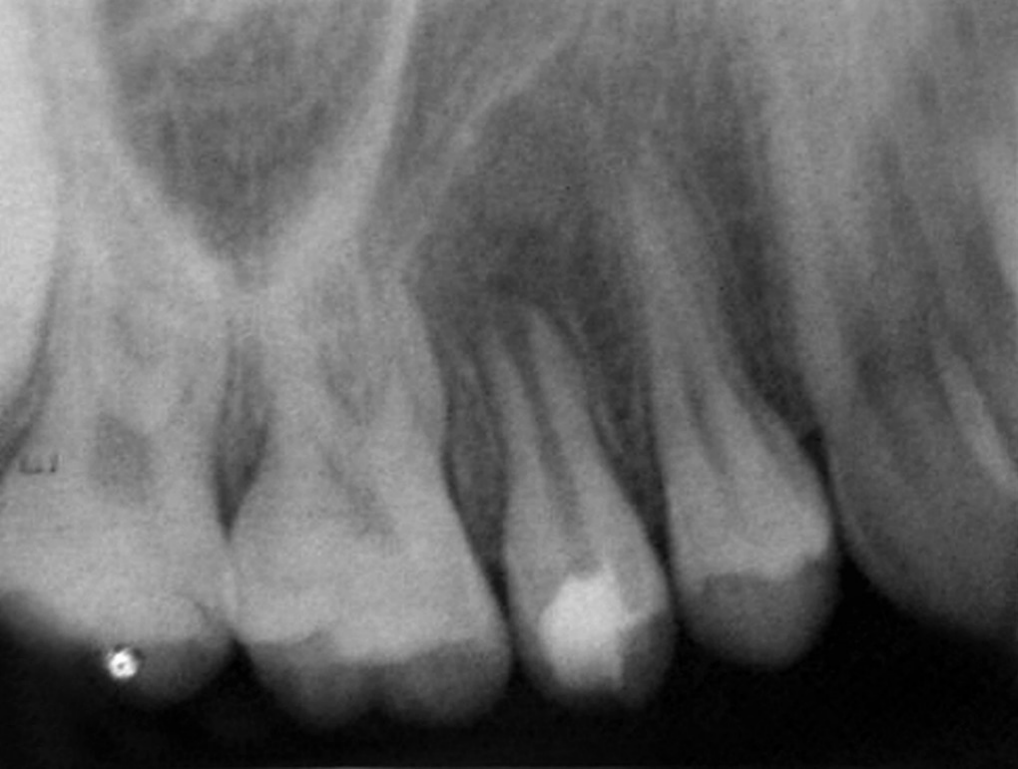
IOPA showing calcium hydroxide dressing in relation to 25

The caries lesion of 25 was accessed with the help of round bur (Fig. 5). Access opening was performed (Fig. 6), necrotic pulp was extirpated, working length was taken, which measured as 14.5 millimeter (Fig. 7). Biomechanical preparation was done. Apexification was done with calcium hydroxide (Fig. 8). The tooth is under observation, once radiographic barrier is formed conventional root canal treatment will be performed.

## DISCUSSION

Dental caries is a multifactorial disease. It is the most prevalent chronic disease. It does not fall into any of well-recognized pathological classification of disease. It is a local disease, which involves destruction of hard tissue of teeth by metabolites produced by oral microorganisms. Dental caries is unique not only in terms of pathological mechanism, but also is a biosocial disease rooted in technology and economy of our society. As living standards improve, severity of disease usually increases.^2^

Caries was earlier defined as microbial disease of the calcified tissue of teeth characterized by the demineralization of inorganic portion and dissolution of organic substance of tooth. Now this definition is updated by International consensus workshop on caries clinical trial-2004, where in caries is a defined process involving an imbalance in the normal molecular interaction between the tooth surface, subsurface or the adjacent microbial biofilm.^3^

Occult/hidden caries is defined as occlusal caries, which is not diagnosed clinically because the occlusal surface appears to be ostensibly intact and shows radiolucencies in dentin.^1^

Prevalence of occult caries

**Table:** 

*Authors/Year*		*Mean age of subjects (N)*		*Teeth*		*Percent occult caries*	
Sawle and Andlaw/1988^4^		14-16 years (740 subjects, 1974)		All 1^st^ and 2^nd^ molars		3.6 (1974)	
		(1319 subjects, 1982)				3.1 (1982)	
Creanor et al/1990^5^		14-15 yrs (2623 subjects)		Mandibular 1^st^ and 2^nd^ molars		11.8	
				Maxillary 1^st^ and 2^nd^ molars,		3.1	
				premolars		0.8	
Kidd et al/1992^6^		Adolescents (6110 teeth)		Mandibular 1^st^ and 2^nd^ molars		12.9	
				Maxillary 1^st^ and 2^nd^ molars		6.3	
Weerheijm et al/1992^7^		14 yrs (131 subjects)				26	
		17 yrs (123 subjects)		All 1^st^ and 2^nd^ molars		38	
		20 yrs				50	
Weerheijm et al/1992 ^8^		12.4 yrs (359 subjects)		All 1^st^ and 2^nd^ molars		15	

## PATHOLOGY

The area of initiation of occult lesions may provide an explanation for the difficulty in their detection. In general, it is thought that occlusal lesions may begin in two locations. The first is an area superficially at or near the entrance to the fissure, where dietary substrates are readily available. The second is on the walls of the fissure near its base, and hidden from direct view. Although, it is most likely that cariogenic substrates reach the bacteria in these locations via penetration through the occlusal fissures, there is speculation that the cariogenic bacteria may be nourished by pulpal tissue fluids present in dentinal tubules. This process would allow cariogenic bacteria to persist in deep, clinically obscured lesions enabling the caries process to continue.^9^

Weerheijm et al (1990) in their microbiological study reported that the bacteria profile within occult lesions was mainly limited to mutans streptococci and lactobacilli, which suggests that these lesions are not associated with microorganisms different to those found in other carious lesions.^9^

Along with occult caries, there is one more entity named as preeruptive intracoronal resorptive lesions. It was reported in a few studies of occult caries that when previous radiographs of the affected teeth during their unerupted stages were examined, they showed that the radiolucencies had been already present in the same locations within the teeth even prior to tooth eruption.^10-13^ These findings suggest that a percentage of occult caries may have their origins as detectable only with the use of radiographs. These defects, present on unerupted teeth, are referred to as intracoronal resorptive defects, and usually detected incidentally on routine dental radiographs. They are often found within the dentine, adjacent to the amelodentinal junction in the occlusal aspects of the crown. Nearly half of the lesions are located on the central aspects of the crown with smaller percentages in mesial or distal aspects of the occlusal surface. In prevalence studies, at the time of discovery, the majority of defects were found to be less than one-third the thickness of dentin. As the lesions resemble caries, they are often referred to as “pre-eruptive caries.”^10-13^ There is little scientific basis for this nomenclature as a pre-eruptive, developing tooth, which is completely encased in its crypt, and is not likely to be infected with cariogenic microorganisms.

To date, clinical and histological evidence substantiates the hypothesis that these defects are acquired as a result of coronal resorption. In the pre-eruptive state, these lesions were reported to contain soft tissue when examined during surgical exposure. Histological examination often reveals signs of resorption, such as scalloping of the lesion margins, as well as resorptive cells is thought to enter the dentin through poorly coalesced enamel fissures or the cemento-enamel junction. Although trigger factors for the resorption are unknown, a high association of ectopic positioning of affected teeth was reported in controlled studies, which suggests that abnormal local pressure may be an inciting factor for the resorption.^10-13^

Although an intracoronal resorptive lesion is unlikely to contain microorganisms in the preemptive stages, once it has emerged into the oral cavity, it rapidly becomes colonized by the oral flora. The retentive nature of the cavitated lesion favors the development of caries, and the lesion becomes indistinguishable from a carious lesion once it is exposed in the oral cavity.^10-13^

Occult lesions, which are undiagnosed may progress rapidly with severe destruction of the crown and endodontic involvement. In the case of pre-eruptive intracoronal resorption lesion, surgical exposure of the developing crown may be necessary, if there is a rapid rate of progress of the lesion. However, many lesions may enlarge only minimally in the pre-eruptive stages, so that it may be possible to wait for tooth emergence before restoration.

### Diagnosis of Occult Lesion

As occult caries is considered to be dentinal caries, which is not diagnosed on visual examination of the occlusal surface but is present on a radiograph of the tooth, the accuracy of diagnosis of the occlusal fissures would clearly play an important role in determining its prevalence.

Diagnosis of caries in occlusal fissures would depend, in turn, on the clinical criteria, which are followed for diagnosis of caries as well as the techniques or instruments used. With regard to diagnostic criteria, the current WHO clinical system has been most commonly employed by clinicians. This system, which considers caries to be present when there is frank cavitation detected by visual inspection or probing, is likely to underdiagnose some occlusal lesions. For example, in the study of Weerheijm et al (1992), it was found that 50% of occlusal surfaces thought to be clinically sound were shown to have dentinal caries when the teeth were later examined histologically.^14^

Bitewing radiographs are one of the most useful aids in the diagnosis of early fissure caries, and their use in conjunction with careful clinical examination techniques are likely to detect occlusal caries efficiently. On the other hand, panoramic radiographs are useful in the detection of intracoronal resorptive lesions in unerupted teeth. It is recommended that the crowns of all unerupted teeth be examined on panoramic radiographs for these lesions.^15^

In addition to clinical criteria, the instruments used may affect diagnostic accuracy as these have different sensitivities and specificities for occlusal caries. In addition to visual and tactile techniques, several noninvasive instruments may be used as adjuncts to aid diagnosis of occlusal caries. Radiography is commonly used by clinicians and has been shown to be of considerable value when visual findings are inconclusive. Other instruments to aid occlusal caries diagnosis include Diagnodent Fibre-optic Transillumination (FOTI), laser luminescence, light scattering, Electrical Resistance Measurements (ERM), and dye uptake.^15^

To conclude, occult caries refers to lesions, which result from inadequate clinical diagnosis, and could have resulted from processes, which were pre-eruptive or post-eruptive. The pre-existence of a pre-eruptive intracoronal resorptive defect may occur in many occult lesions. Upon eruption of the teeth, pre-eruptive lesions become indistinguishable from those resulting from true fissure caries. The prevalence of occult lesions in a community will depend on differences in operator ability to diagnose caries and the criteria applied for caries diagnosis, as well as the examination techniques used. Early diagnosis of occult lesions is the best management. As radiographs are probably the most effective method of diagnosing all occult lesions, they should be recommended at appropriate ages to aid early detection of these lesions. Also, examination of the crowns of unerupted teeth for intracoronal defects is suggested on all routine radiographs.
